# Primary Biliary Acids Inhibit Hepatitis D Virus (HDV) Entry into Human Hepatoma Cells Expressing the Sodium-Taurocholate Cotransporting Polypeptide (NTCP)

**DOI:** 10.1371/journal.pone.0117152

**Published:** 2015-02-03

**Authors:** Isabel Veloso Alves Pereira, Bettina Buchmann, Lisa Sandmann, Kathrin Sprinzl, Verena Schlaphoff, Katinka Döhner, Florian Vondran, Christoph Sarrazin, Michael P. Manns, Cláudia Pinto Marques Souza de Oliveira, Beate Sodeik, Sandra Ciesek, Thomas von Hahn

**Affiliations:** 1 Institut für Molekularbiologie, Medizinische Hochschule Hannover, Hannover, Germany; 2 University of São Paulo School of Medicine, Department of Gastroenterology, Clinical Division, Hepatology Branch (LIM-07), Sao Paulo, Brazil; 3 Klinik für Gastroenterologie, Hepatologie und Endokrinologie, Medizinische Hochschule Hannover, Hannover, Germany; 4 Medizinische Klinik 1, Universitätsklinikum Frankfurt, Frankfurt, Germany; 5 Institut für Virologie, Medizinische Hochschule Hannover, Hannover, Germany; 6 Klinik für Allgemein-, Viszeral- und Transplantationschirurgie, Medizinische Hochschule Hannover, Hannover, Germany; Inserm, U1052, UMR 5286, FRANCE

## Abstract

**Background:**

The sodium-taurocholate cotransporting polypeptide (NTCP) is both a key bile acid (BA) transporter mediating uptake of BA into hepatocytes and an essential receptor for hepatitis B virus (HBV) and hepatitis D virus (HDV). In this study we aimed to characterize to what extent and through what mechanism BA affect HDV cell entry.

**Methods:**

HuH-7 cells stably expressing NTCP (HuH-7/NTCP) and primary human hepatocytes (PHH) were infected with *in vitro* generated HDV particles. Infectivity in the absence or presence of compounds was assessed using immunofluorescence staining for HDV antigen, standard 50% tissue culture infectious dose (TCID50) assays and quantitative PCR.

**Results:**

Addition of primary conjugated and unconjugated BA resulted in a dose dependent reduction in the number of infected cells while secondary, tertiary and synthetic BA had a lesser effect. This effect was observed both in HuH-7/NTCP and in PHH. Other replication cycle steps such as replication and particle assembly and release were unaffected. Moreover, inhibitory BA competed with a fragment from the large HBV envelope protein for binding to NTCP-expressing cells. Conversely, the sodium/BA-cotransporter function of NTCP seemed not to be required for HDV infection since infection was similar in the presence or absence of a sodium gradient across the plasma membrane. When chenodeoxycolic acid (15 mg per kg body weight) was administered to three chronically HDV infected individuals over a period of up to 16 days there was no change in serum HDV RNA.

**Conclusions:**

Primary BA inhibit NTCP-mediated HDV entry into hepatocytes suggesting that modulation of the BA pool may affect HDV infection of hepatocytes.

## Introduction

Chronic infection with hepatitis B virus (HBV) affects over 300 million individuals worldwide [1, 2]. Of these over 15 million are thought to be co-infected with hepatitis D virus (HDV) [1]. For HBV infection several oral drugs are available that efficiently suppress viral replication and can avert most of the morbidity and mortality associated with the disease [3]. These agents however are inactive against HDV replication. The only available treatment option for HDV infection is thus interferon alpha. Interferon treatment clears HDV infection in less than 20% of cases given that a significant number of individuals that are HDV RNA negative at the of treatment and/or 24 weeks later relapse at even later time points [4, 5]. Thus, better options for the treatment of hepatitis D, widely considered more severe than viral hepatitis B alone, are urgently needed.

HDV lacks the ability to synthesize envelope proteins and is dependent on the presence of the HBV envelope proteins to package its genome and produce infectious particles. Given that HBV and HDV both share the same envelope they are thought to enter hepatocytes in the same manner. Recently, the sodium-taurocholate cotransporting polypeptide (NTCP) has been identified as an essential hepatocyte expressed receptor for HBV and HDV [6]. This has lifted a major roadblock for the HBV/HDV field since cell lines engineered to express human NTCP now provide a convenient model system to study the complete HBV and HDV replication cycle *in vitro* [6–8].

Some agents that target NTCP have already been shown to inhibit HBV and/or HDV cell entry. A myristoylated 47 amino acid fragment from the preS1 region of the large HBV envelope protein (preS1 peptide) binds NTCP and acts as a potent competitive inhibitor of viral entry [6, 7, 9]. PreS1 peptide is being developed as an anti-viral therapeutic under the name Myrcludex and has entered clinical trials [10]. Cyclosporine A, an immunosuppressant drug known to cause cholestasis as a side effect, has recently been shown to inhibit HBV entry most likely by competing for a common binding site on NTCP [11, 12]. Finally, taurocholate, the prototypical substrate of NTCP has been reported to inhibit HBV entry to some extent [11]. Very recently, a number of other biliary acids have also been reported to inhibit HBV and HDV entry most likely because they compete with the large HBV surface protein for a partially overlapping binding site on the NTCP molecule [8]. In this study we aimed to better characterize and quantify the effects of different groups of BA on HDV cell entry *in vitro* and assess their effect on other replication cycle stages. Moreover, we report on three cases where chenodeoxycholate that is licensed in Germany for the treatment of gallstones was administered to individuals with chronic HBV/HDV coinfection.

## Materials and Methods

### Antibodies, biliary acids and drugs

Chenodeoxycholic acid (CDCA; C9377), cholic acid (CA; C1129), dehydrocholic acid (DHCA; 30830), deoxycholic acid (DCA; D2510), glycocholic acid (GCA; G2878), lithocholic acid (LCA; L6250), sodium chenodeoxycholate (SCDCA; C8261), sodium glycochenodeoxycholate (SGCDCA; G0759), sodium taurocholate (STCA; T4009), sodium taurodeoxycholate (T0875), and ursodeoxycholic acid (UDCA; U5127) were purchased from Sigma (Germany). Cyclosporine A (CsA) was purchased from Toronto Research Chemicals (Canada). The myristoylated 47 amino acid preS1 fragment derived from the large HBV envelope protein (preS1 peptide) as well as an atto 488-labeled version and an atto 488-labeled mutant version unable to bind to NTCP were a kind gift from Stephan Urban (University Heidelberg).

A polyclonal anti-NTCP antibody [13] was kindly provided by Bruno Stieger (Universitätsspital Zurich, Switzerland); polyclonal anti-HDV was kindly provided by John Taylor (Fox Chase Cancer Center, Philadelphia, USA).

### DNA constructs

The open reading frame of human NTCP was synthesized by Eurofins MWG (Ebersberg, Germany), cut out by restriction digest and ligated into the lentiviral blasticidin selectable pWPI/Bsd vector. Transgene expression in pWPI-Bsd is driven from a CMV promoter. The correct sequence of pWPI-Bsd/hNTCP was confirmed by DNA sequencing. The plasmids pSVLD3 encoding a trimer of the HDV genome [14] and pT7HB2.7 encoding the HBV preS1, PreS2 and S genes were a kind gift from C. Sureau (Laboratoire de Virologie Moleculaire, INTS, France).

### Cell lines and cell culture

We established an HuH-7 derived cell line stably overexpressing human NTCP (HuH-7/hNTCP). All cells were maintained in Dulbecco’s modified eagle medium (DMEM; Invitrogen, Karlsruhe, Germany) supplemented with 10% fetal calf serum (FCS), L-glutamine, nonessential amino acids, penicillin and streptomycin (Invitrogen). HuH-7/hNTCP cells were selected with 10 µg/mL of blasticidin (Life Technologies, USA). Isolation of primary human hepatocytes (PHH) was done as before using a modified 2-step collagenase (Roche, Mannheim, Germany) perfusion technique that has previously been described [15]. PHH were kept in William´s medium E (all Biochrom AG, Berlin, Germany) with insulin (1 µM), dexamethasone (1 µM), penicillin/streptomycin, sodium pyruvate, HEPES buffer, L-glutamine and 5% FCS.

### Cytotoxicity and growth inhibition assay

As a global test for effects of biliary acids on cell viability and/or proliferation we used HuH-7 or HuH-7/hNTCP cells stably expressing firefly luciferase under the control of a CMV promoter as previously described [16]. Cells were exposed to biliary acids and kept in culture for the same duration as in the respective infection or replication/particle release experiments performed in parallel. Then cells were lyzed and luciferase activity was measured.

### Production of HDV particles and replication assay

To produce HDV particles we used the method described by Sureau et al. [17]. 8x10^5^ HuH-7 cells per well were seeded in a 6 well plate. The next day a mixture of 1.65 µg of pSVLD3 and 1.65 µg of pT7HB2.7 per well were mixed with 153 µL OptiMEM (Life Technologies, USA) and 12 µL of FuGENE HD (Promega), incubated for 15 minutes at room temperature and 150 µL were added to the cells. Media was changed after 16 hours and then every other day until supernatant from transfected cells was collected on days 7, 9, 11 after transfection. The supernatant was filtered (0.45 µM) and then used to infect the HuH-7/hNTCP cells as described below.

To measure HDV particle assembly and release, we used 5x10^4^ cells per well in a 12 well plate. The transfection mixture was scaled down in proportion to well surface area. The day after transfection BA were added at the desired concentration. After 7 days we collected the supernatant to infect HuH-7/hNTCP (target) cells to check for the release of infectious particles. In parallel the transfected (producer) cells were fixed and stained for HDV antigen.

HDV replication in presence of BA was tested by infecting 2x10^4^ HuH-7/hNTCP cells in a 12-well plate with HDV. After 6 h, the virus was removed and DMEM (3% FCS) with or without BA at desired concentrations were added. Medium with or without BA was replenished after 3 days. On day 7, cells were fixed for RNA preparation and q-RT-PCR was used as readout. Assembly and replication experiments were done in triplicates.

### Infection assays

For infection experiments supernatants containing HDV particles were supplemented with PEG8000 to a final concentration of 4%. BA or Myrcludex or CsA or DMSO were added as needed. Cells were seeded at 3x10^4^ cells per well in a 24-well plate and at 1x10^4^ cells per well in a 96-well plate for immunofluorescence (IF) and immunohistochemistry (IHC) / 50% tissue culture infectious dose (TCID_50_) assays, respectively. Cells were exposed to virus at a multiplicity of infection of 0.5 or less for 6 hours then media was exchanged and the cells were incubated for another 5 days in the absence of PEG, BA or drugs. Cells were then washed and fixed with 3% paraformaldehyde for 20 min for IF and in ice cold methanol for IHC, washed again with 1xPBS and permeabilized for 30 min with 0.2% Triton X-100 in PBS (IF) and for 20 min with 0.5% Triton X-100 in PBS (IHC). After being washed with PBS, cells were blocked with 1% bovine serum albumin (BSA) for 1 hour and then incubated with anti-HDV diluted 1:1000 in PBS/0.5% BSA with agitation for 1 hour. Subsequently, cells were washed with PBS three times before being exposed to the secondary antibody for 1 hour with agitation. Secondary antibodies were Alexa Fluor 488 goat anti-Rabbit IgG at 1:1000 (Life Technologies) and anti-rabbit horse radish peroxidase at 1:200 in 0.5% BSA (Sigma, Germany), for IF and IHC/TCID_50_, respectively. Cells were again washed 3 times. IHC was performed at 4°C and IF at room temperature. When performing IF cells were counterstained with 4,6-diamidino-2-phenylindole (DAPI; Life Technologies) and images were obtained by fluorescent microscopy (Leica DM6000). Alternatively, we imaged nuclei and HDV positive cells from 18 independent sites within two separate wells using a wide-field high content fluorescence microscope fitted with a 10x objective (ImageXpress Micro, Molecular Devices, Biberach an der Riss, Germany). In IHC assays HRP activity was visualized with homemade chromogenic substrate composed of 5 mL sodium acetate/acetic acid mixture (37.5 µM / 15 µM), 1.5 mL carbazole (0.124 mM) in nn-dimethylformamide, 20 µL H_2_O_2_. The reaction was stopped with deionized water.

For infection in low extracellular sodium conditions HDV containing supernatant was loaded on an Amicon Ultra 15 mL filter system and washed multiple times with either low sodium solution (145 mM KCl; 5 mM NaCl; 0.25 mM CaCl_2_; 10 mM HEPES; 10 mM D-glucose [pH adjusted to 7.4]) or regular sodium solution (5 mM KCl; 145 mM NaCl; 0.25 mM CaCl_2_; 10 mM HEPES; 10 mM glucose [pH adjusted to 7.4]).

To determine TCID_50_ cells in a 96-well plate were infected with the virus in serial dilutions from 10^-0.3^ to 10^-8^ in presence or absence of BA or Myrcludex with six wells for each dilution. Cells were maintained for five days and then fixed and stained for IHC. TCID_50_ was calculated according to the methods described by Spearman and Kärber [18, 19]. Briefly, HDV containing supernatant was diluted in 8 five-fold serial dilution steps and 6 wells were scored per dilution so that each TCID_50_ data point is based on a total of 48 individual wells. All infection experiments were repeated at least three times on different days.

### Quantitative PCR

HDV RNA was quantified by a validated in-house quantitative PCR (qPCR) assay in use for standard clinical care at Hannover Medical School and University Hospital Frankfurt. A Lightcycler 480 II (Roche Diagnostics, Indianapolis, IN, USA) was used. Details on the assay have been published [20]. For tissue culture experiments SYBR green based quantification of GAPDH was used to control for cell number.

### Binding assay

For binding competition assay HuH-7/hNTCP cells were trypsinized. Then trypsination was stopped by adding 1 mL FACS buffer (PBS / 2% BSA / 0.02% NaN_3_) and cells were centrifuged at 300 rcf for 5 minutes at room temperature to remove buffer. Cells were resuspended in FACS buffer with or without BA and incubated on ice for 15 minutes before 100 µL of staining buffer (FACS buffer + 200 nM atto 488-labeled preS1 peptide kindly provided by Stephan Urban, Heidelberg, Germany) were added. Cells were then incubated for another 10 min on ice. The cells were washed 3 times and resuspended in FACS buffer. Fluorescence intensity was quantified using a FACSCanto cytometer (Becton Dickinson, BD, USA) and data were analyzed with FlowJo software (Tree Star, Ashland/OR, USA).

### Patient sera and ethics

We report a summary of three cases of chronic HBV/HDV coinfection where chenodeoxycholic was administered. Chenodeoxycholic acid is licensed for the treatment of gallstone disease. Ethics comitee approval was not necessary since off label administration of licensed drugs for other indications as an individualized treatment attempt (“*Individueller Heilversuch*”) is admissible in Germany without review by an ethics committee. Patients were given the drug as an individualized off label treatment attempt by two of the authors (KS and CS). These authors did so in their capacity as treating physicians of the patients. All individuals are regular patients at the Frankfurt University Hospital liver clinic where they are regularly seen and receive care for chronic hepatitis B/D. The three patients were two males (31 and 52 years old) and one female (43 years old). All had Child Pugh Stage A cirrhosis. The off label use of chenodesoxycholate as well as expected risks and benefits were discussed in detail with all three individuals. Informed consent was obtained verbally and documented in the patient chart. Serum HDV RNA was determined before the first dose of chenodeoxycholic acid and at several time points thereafter. When viral load remained unchanged the drug was discontinued after 7 to 16 days.

The cell line established as part of this work (HuH-7/hNTCP) is a derivative of the HuH-7 cell line that was originally established in Japan in 1982 [21]. HuH-7 cells have since been widely used by many laboratories for numerous studies. We obtained an aliquot from C. Rice (The Rockefeller University, USA). None of the authors were involved in collecting these cells.

### Statistical analyses

Where required unpaired t-test or linear regression were performed as appropriate to test for statistical significance. p-values below 0.05 were considered significant.

## Results

To have a robust system to perform HDV infection *in vitro* the open reading frame of human NTCP was synthesized, cloned into the blasticidin selectable pWPI-Bla lentigenome and stably expressed in HuH-7 cells producing a homogenous cell population. IF staining confirmed expression of NTCP in all or almost all cells in the HuH-7/hNTCP population (S1 Fig.). By FACS 96.4% of cells stained positive (S1 Fig.). Thus, we conclude that over 95% of cells were NTCP-positive.

**Fig 1:**
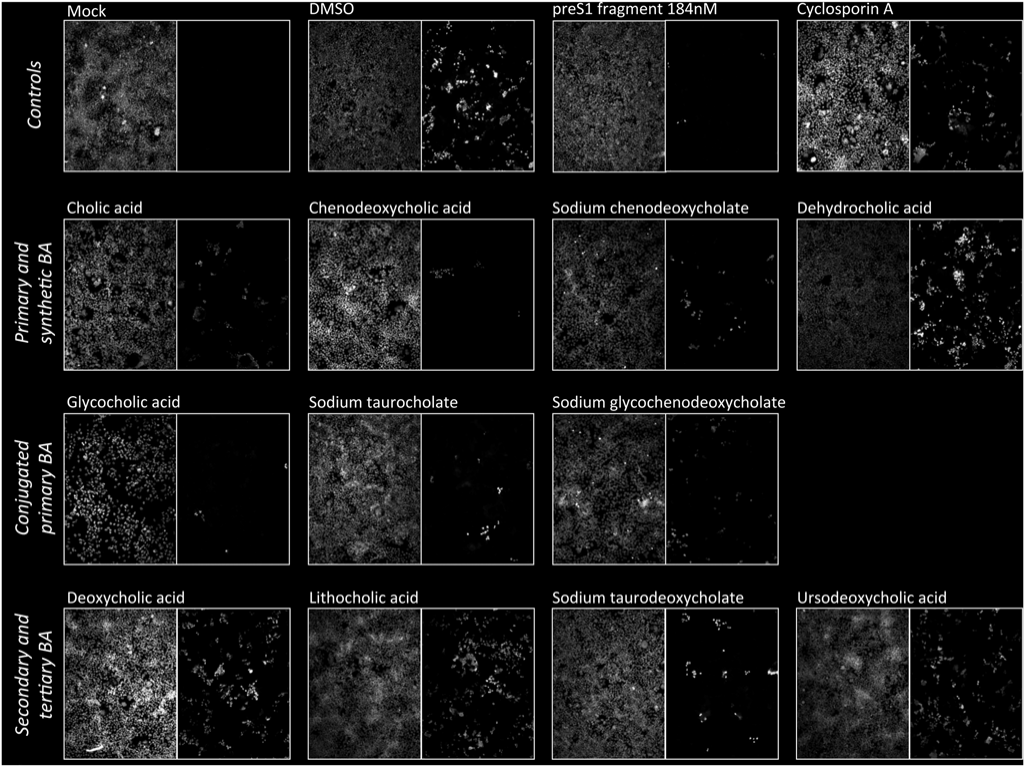
Effect of BA on HDV infection of HuH-7/hNTCP cells. HuH-7/hNTCP cells were exposed to HDV containing supernatant in the presence or absence of 200 µM of different BA. For glycocholic acid 50 µM results are shown because the BA is toxic at 200 µM. preS1 peptide (368 nM) and cyclosporine A (25 µg/mL) served as positive controls. After 6 h cells were washed and repleted with regular media without virus, BA or drugs. After another 5 d cells were fixed and stained with a polyclonal antibody against HDV antigen (right hand images in each panel). Nuclei were counterstained with DAPI (left hand images). A representative of at least three independent experiments is shown.

**Fig 2:**
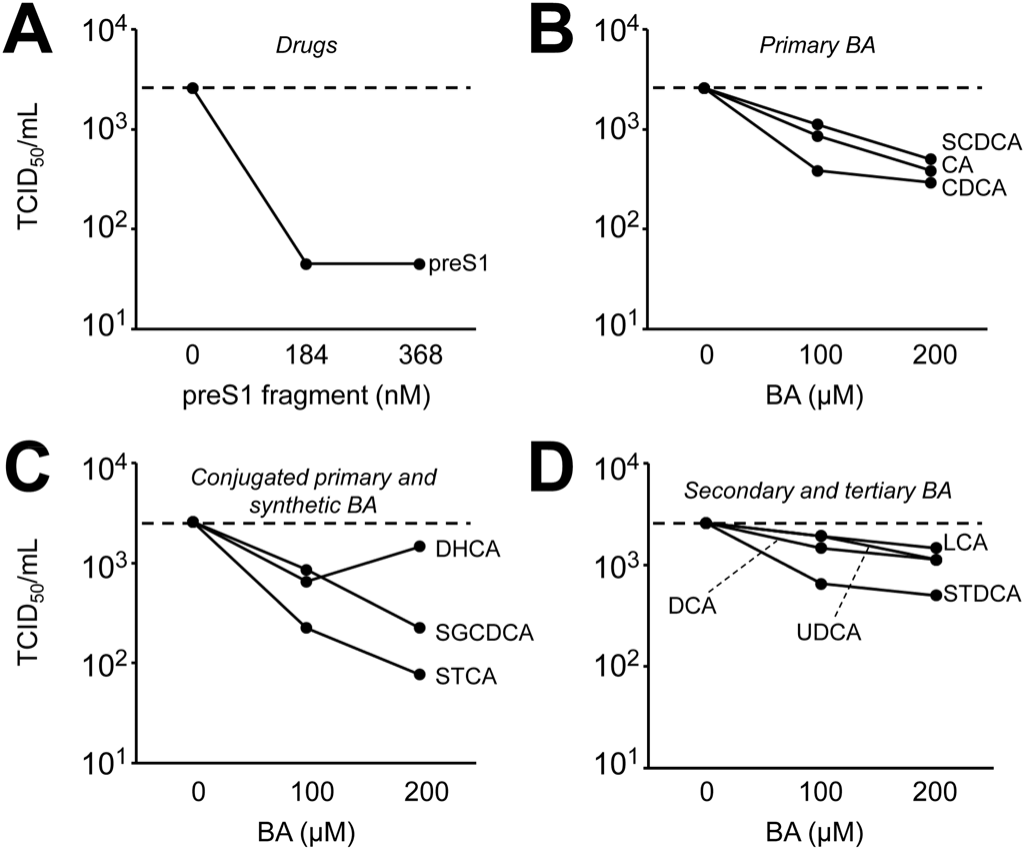
Quantification of inhibition of HDV cell entry by BA. HuH-7/hNTCP cells were seeded in 96-well plates and infected with HDV in the presence of **(A)** 184 or 368 nM or preS1 peptide or **(B-D)** 100 or 200 µM of BA. Cells were exposed to virus +/- BA for 6 h, washed and then kept in regular media for another 5 days. TCID_50_ values were determined as described in the method section. Each data point is based on 48 wells and a representative of two independent experiments is shown.

HuH-7/hNTCP cells were exposed to supernatant containing infectious HDV for six hours after which media was exchanged. Cells were incubated for another 5 days and then fixed and stained. A large percentage of cells stained positive for HDV antigen (Fig. 1). HDV antigen positive cells were not observed when HuH-7/hNTCP were mock infected or when parental HuH-7 cells were exposed to HDV (Fig. 1 and data not shown). As expected, addition of preS1 peptide also efficiently blocked infection [9]. Addition of cyclosporine A, a known blocker of NTCP and NTCP-mediated HBV cell entry [11, 12], had a moderate effect. When BA at a concentration of 200 µM were added during the initial 6 hours of infection a variable reduction in the number of HDV antigen positive cells was observed with an apparently more pronounced effect for primary BA (Fig. 1). A subset of BA was also tested by qPCR with comparable results (S2 Fig.). Likewise, when a subset of BA was tested for inhibitory effects on HDV infection of PHH all BA with the exception of the synthetic BA dehydrocholic acid potently inhibited HDV infection (S3 Fig.). To better quantify the reduction in HDV infectivity by BA we performed TCID_50_ assays in the presence of increasing concentrations of preS1 peptide as a positive control (Fig. 2A) or different BA (Fig. 2B-D). We observed some reduction in infectivity by all BA with a more marked effect seen with conjugated and unconjugated primary BA (range 81–97% and mean 89% reduction in TCID_50_/ml at 200 µM of BA compared to infection in the absence of BA) compared to other—secondary, tertiary and synthetic—BA (range 44–81%, mean 56%) (p = 0.002 for comparison of reduction in TCID_50_ at 200 µM of primary BA versus other BA).

**Fig 3:**
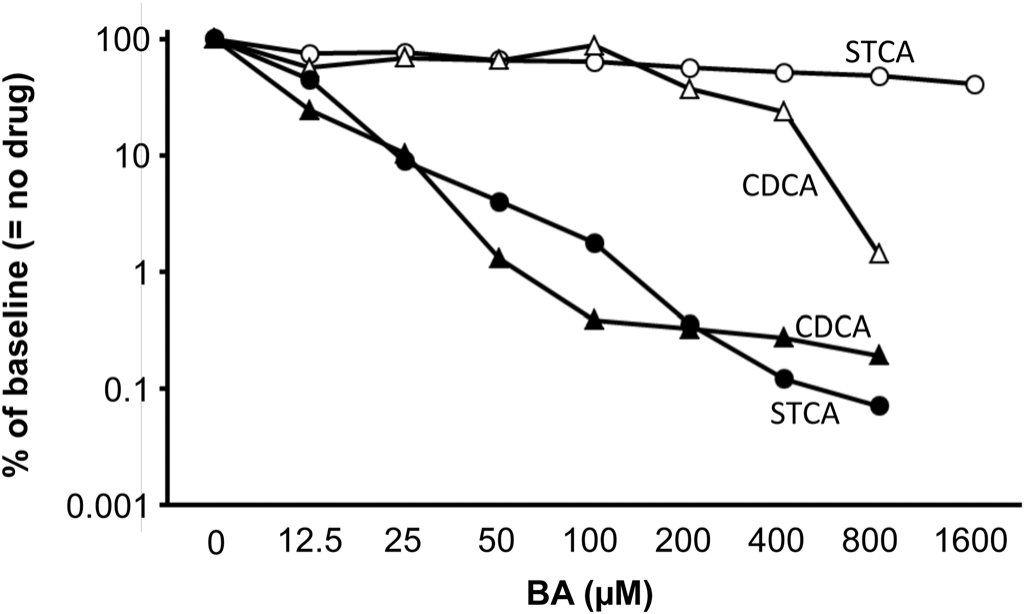
Inhibition-toxicity relationship for chenodeoxycholic acid and sodium taurocholate. To assay HDV infectivity (filled symbols) HuH-7/hNTCP cells were infected as described above in a 96-well plate and TCID_50_ was performed in the presence of increasing concentrations of chenodeoxycholic acid (CDCA; triangles) or sodium taurocholate (STCA; circles). In parallel, using identical concentrations and duration of exposure HuH-7/hNTCP cells stably expressing firefly luciferase were treated with CDCA or STCA and luciferase activity after drug treatment was measured as an aggregate measure of cytotoxic and anti-proliferative effects (open symbols). A representative of three independent experiments (only two for cytotoxicity) is shown. Each data point is based on analysis of 48 wells in a TCID_50_ setup.

Subsequently, STCA, the BA with the most pronounced inhibition of HDV infectivity in the initial experiments, and CDCA that is available as an approved drug were examined in more detail. Both are primary BA. Performing TCID_50_ assays over a wider concentration range we observed a dose dependent up to 1000-fold inhibition of HDV infectivity (Fig. 3). However, even at the highest concentration tested (800 µM) there was still residual infectivity. In parallel, HuH-7/hNTCP cells stably expressing firefly luciferase were exposed to the same concentration of BA for the same duration and luciferase activity was measured as an aggregate measure of cell viability and proliferation. At STCA concentration of 800 µM the signal was reduced by 52% suggesting that the >99.9% reduction in infectivity seen at this high BA concentration was mostly due to inhibition of HDV infection and not to effects of BA on cell growth and/or cell viability. CDCA was somewhat more toxic showing a 63% reduction of viability and 99.7% reduction of infectivity at 200 µM.

Expression of HDV antigen appeared unaltered when two DNA plasmids encoding the HDV genome and the HBV envelope proteins were transfected into HuH-7 cells and cells were then cultured for 7 days in the presence or absence of different BA (S4 Fig.). Day 7 was chosen, because around this time after transfection cellular HDV RNA approaches a plateau (data not shown).

The replication process of HDV also seems unaltered in presence of BA. When HuH-7/hNTCP cells were infected with HDV and after removal of the virus, BA at 100 µM (GCA and SGCDCA at 50 µM) were applied for 7 days, cellular HDV RNA as measured by qPCR was not significantly different in the presence of any of the BA tested with exception of the primary BA SCDCA and the secondary BA LCA (S5 Fig.). This suggests that intracellular HDV replication is unaffected by the presence of BA and that the inhibitory effect observed when infection is performed in the presence of BA is indeed due to a block at the cell entry stage of the HDV replication cycle. Then supernatant from cells cotransfected with the HDV genome and the HBV envelope genes and cultured in the presence or absence of BA was harvested, PEG precipitated and used to infect naïve HuH-7/hNTCP cells in the absence of BA. No apparent differences in the number of HDV antigen positive cells was noted again suggesting that the particle assembly and release stage of the HDV replication cycle is unaffected by BA (S6 Fig.).

**Fig 4:**
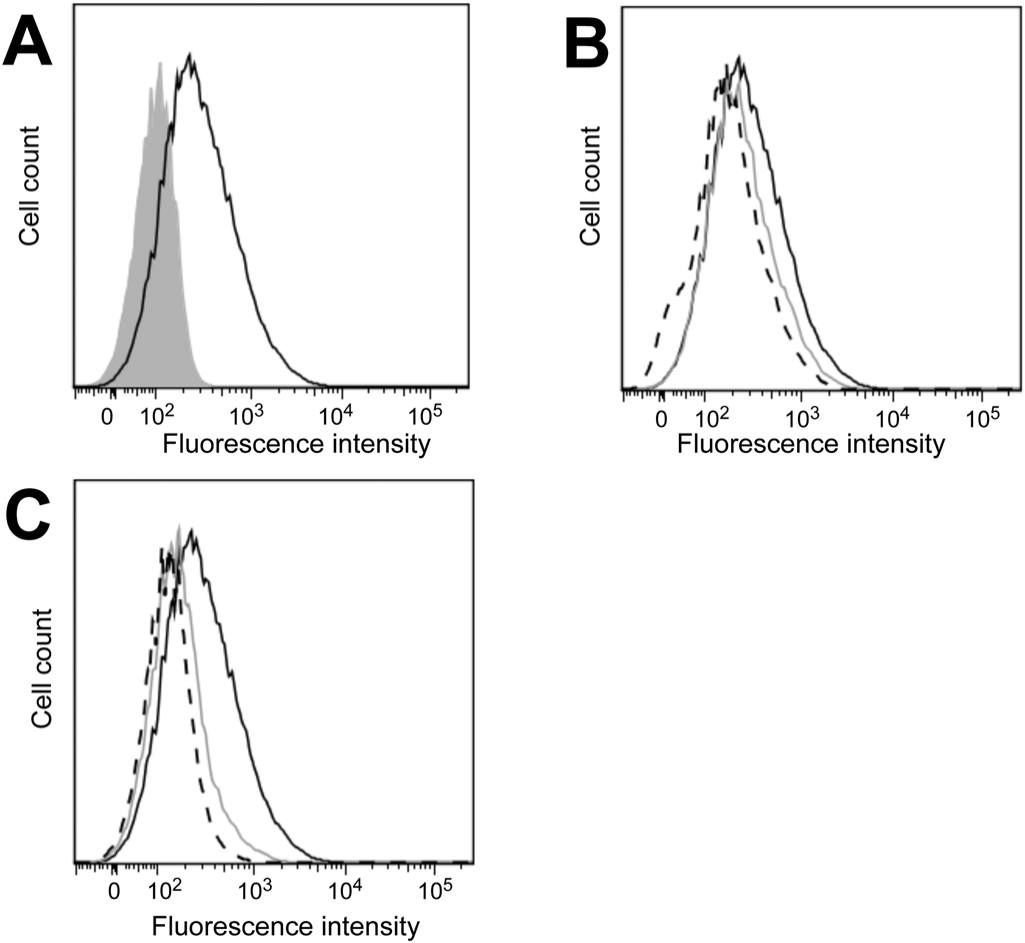
Binding of BA to NTCP. **(A)** Binding of atto 488-labelled preS1 (black line histogram) and a mutant version unable to bind to NTCP (grey shaded histogram) to HuH-7/hNTCP cells was assayed by flow cytometry. **(B+C)** Binding of Atto 488-labelled preS1 was quantified in the absence (solid black line) of BA or in the presence of 100 (grey line) and 200 (dashed black line) µM of (B) lithocholic acid or (C) sodium taurocholate.

To address the mechanism of BA-mediated inhibition of HDV we tested the ability of inhibitory BA to compete for large HBV surface protein binding to NTCP expressing HuH-7 cells. Atto-488 labelled preS1 peptide bound to NTCP expressing HuH-7 cells while an Atto-488 labelled mutated version of the peptide without affinity to NTCP did not indicating that binding occurred through a specific interaction (Fig. 4A). The inhibitory BA lithocholic acid (Fig. 4B) and sodium taurocholate (Fig. 4C) showed a concentration dependent reduction in binding of preS1 peptide suggesting that inhibitory BA and the viral surface protein compete for binding to NTCP.

**Fig 5:**
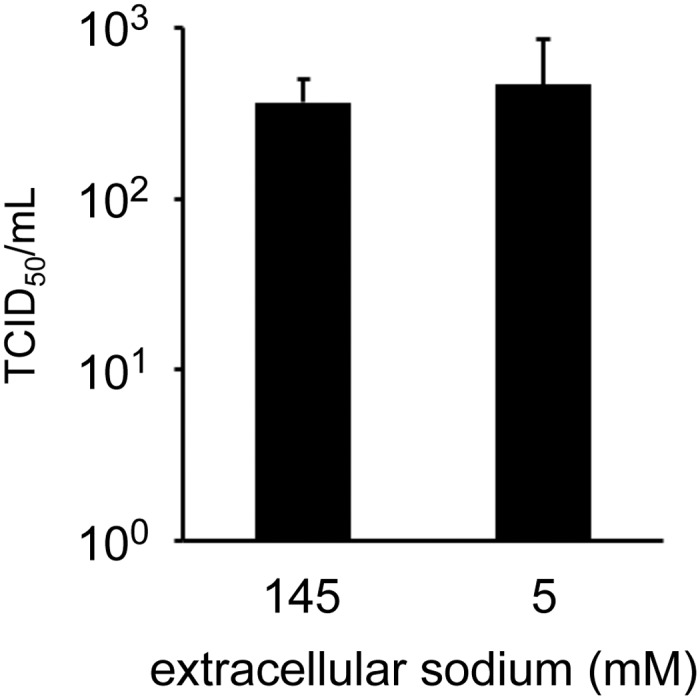
HDV infection in the absence of a sodium gradient. HuH-7/hNTCP cells were exposed to HDV contained in a solution with regular (145 mM Na^+^) or low (5 mM Na^+^) sodium for 6 hours. Then cells were washed and wells were repleted with regular media. TCID_50_ assay was performed on day 5 after infection. Each data point represents the mean +/- SD of n = 4 individual TCID_50_ assays (48 wells each) performed on separate occassions.

NTCP uses the physiological sodium gradient from about 145 mM in the extracellular space to 5–10 mM in the intracellular space to transport BA against a BA concentration gradient into the hepatocyte in a secondary active manner. To test whether this cotransporter function of NTCP is required for HDV cell entry we performed HDV infection under conditions with regular (145 mM) and low (5 mM) extracellular sodium in a TCID_50_ format. There was no significant difference in HDV titer in the presence or absence of a sodium gradient across the plasma membrane (Fig. 5) arguing against a relevance of the physiological transporter function of NTCP for HDV cell entry.

**Fig 6:**
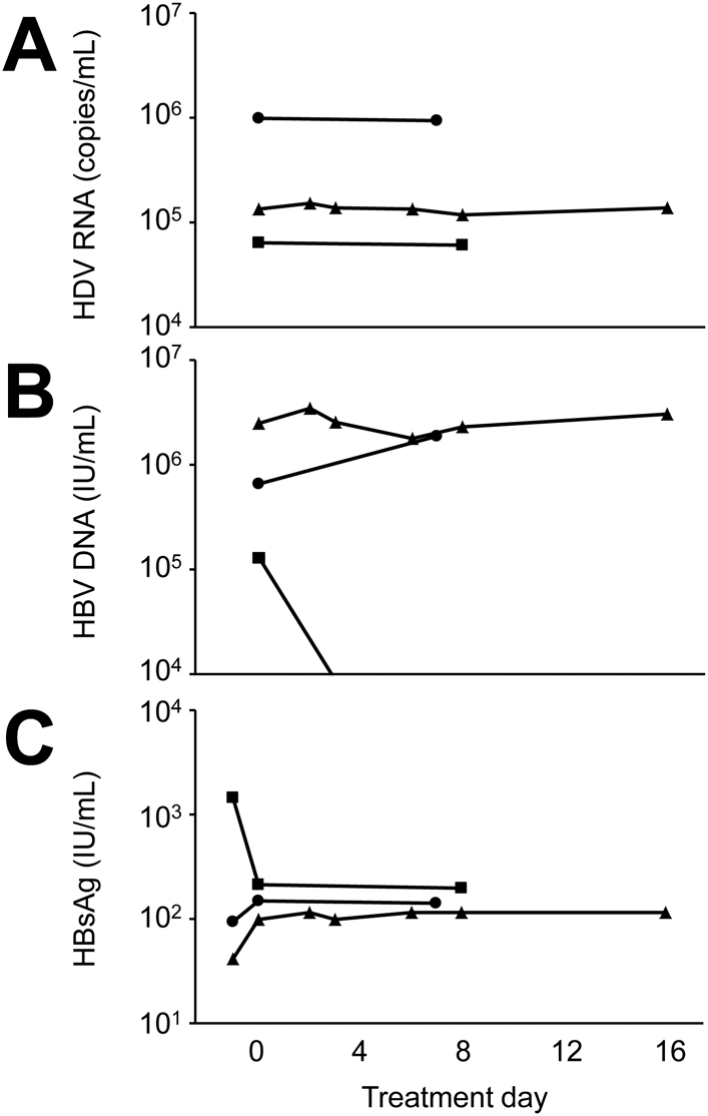
Effect of short-term administration of chenodeoxycholic acid on serum HDV RNA. Three individuals with chronic HBV/HDV coinfection (circle, triangle and square) were administered chenodeoxycholic acid 15 mg/kg daily orally in three doses. (A) Serum HDV RNA, (B) serum HBV DNA and (C) HBsAg were determined before the first dose and at the indicated time points thereafter. The last symbol in each line indicates serum levels after the final dose of chenodeoxycholic acid. HBV DNA for one patient (square) was not detectable on day 8.

Finally, we report three cases of individuals with chronic HBV/HDV coinfection that were administered CDCA at 15 mg/kg orally for 7–16 days. Serum HDV RNA was measured by quantitative PCR before the start, during and at the end of administration of chenodeoxycholic acid (Fig. 6). There was no change in HDV viral load and no adverse events were noted; one patient reported abdominal discomfort and nausea that may have been related to CDCA, whereas the other two experienced no side effects. HBV viral load was low (<10,000 IU/mL) in all cases and there was no apparent change over the period where chenodeoxycholic acid was administered (Fig. 6).

## Discussion

Primary BA are synthesized in hepatocytes where they can also be conjugated with either glycine or taurine producing the more soluble conjugated primary BA [22]. BA are secreted through the biliary tract into the intestine where secondary BA are generated through processing or primary BA by intestinal microorganisms. About 95% of BA are then reabsorbed by the intestine and transported back to the liver via the portal vein where NTCP is the most important factor mediating sodium-dependent uptake of BA from the portal blood into hepatocytes. Tertiary BA are formed from secondary BA either in the intestine or the liver. The total pool of bile salts in adult humans is about 3–4 g and circulates through the enterohepatic circulation about 6–10 times per 24 hours [23]. We found that most BA have some inhibitory effect on HDV cell entry. Primary BA had a more pronounced inhibitory effect compared to other BA groups including secondary, tertiary and synthetic BA. BA competed with a fragment from the large HBV surface protein for binding to NTCP-expressing cells. These findings largely confirm a recently published comprehensive study by Yan and colleagues [8]. Also, while this manuscript was under revision, Koenig and colleagues showed inhibition of HBV entry after preincubation of NTCP-HepG2 cells with BA [24]. We also observed that HDV entry occurs normally in the absence of a sodium gradient across the cell membrane suggesting that the transport function of NTCP that uses the sodium gradient to drive BA transport is not required for infection. Thus our data and data published by others are more consistent with direct competition of the incoming virion with BA for a common or at least overlapping binding site on the NTCP molecule. The role of the NTCP co-transporter function in HBV or HDV cell entry had not been previously investigated to our knowledge.

Differently from the very recently published work by Yan and colleagues [8] we used a direct measure of infectivity (TCID_50_) and observed a more pronounced inhibitory effect of primary compared to secondary, tertiary and synthetic BA tested. Moreover, we demonstrate that both other replication cycle stages, replication and assembly/release, are largely unaffected. The most important difference to previously published work may be that in our hands the full inhibitory potential of BA is apparent only in a clearly supraphysiological concentration range: as shown in Fig. 3 we observed a more than 90% reduction in TCID_50_ at 25 µM and more of sodium taurocholate. While the physiological concentration of total BA in human serum is 1–6 µM it can be elevated to more than 100-fold the upper limit of normal under conditions of intra—or extrahepatic biliary obstruction [25–27]. While such high serum BA levels are often associated with pruritus they are not by themselves toxic. Conversely, in the work by Yan and colleagues 50% inhibition of HBV entry was observed at around 1 μM and more than 90% inhibition at around 10 μM of BA/ STCA. We cannot fully explain this difference, however, there were several experimental differences, most notably the use of HBV versus HDV and HepG2/NTCP versus HuH-7/NTCP hepatoma cells. We think that marked inhibition of viral infection by a physiologically occurring concentration of a BA is somewhat unlikely given the known high infectivity of HBV and HDV *in vivo*, but inhibition might be more likely to occur in common conditions where serum BA are increased such as cirrhosis or post-hepatic cholestasis. Our clinical observation that exogenous administration of chenodeoxycholic acid does not affect HDV viral load at all argues against a major impact of BA on HDV infectivity *in vivo*. In summary, we doubt that BA-mediated inhibition of HDV infectivity plays a major role in the context of human infection. However, BA are still of interest for mechanistical studies of the interaction between HBV or HDV and NTCP such as this paper and the work by Yan and colleagues [8].

As yet there are no approved drugs or compounds in advanced clinical development perturbing the HDV replication cycle. Prenylation inhibitors and the HBV/HDV entry inhibitor Myrcludex are promising approaches, but their clinical potential with regard to HDV cure is as yet unclear [10]. Moreover, as a very small virus encoding only a single protein without obvious enzymatic activity HDV offers no obvious druggable targets. Thus, the discovery of NTCP as an essential HDV receptor [6] and potential drug target is of major significance. The bile acid pool is to some extent amenable to therapeutic modification. A number of smaller clinical studies has examined the use of BA, mostly ursodeoxycholic acid, in patients with chronic hepatitis B or C. A recent meta-analyis concluded that while there seems to be a significant reduction in transaminases associated with administration of BA there was no detectable effect on viral clearance [28]. Thus, whether the observed effect is due to direct anti-viral or rather anti-inflammatory properties of BA is unclear. In Europe, two BA compounds are on the market as drugs: the tertiary BA ursodeoxcholic acid is approved for dissolution of gallstones, primary biliary cirrhosis and symptomatic gastropathy due to biliary reflux. Ursodeoxycholic acid has only a modest inhibitory effect on HDV entry in our hands. Conversely, the primary BA CDCA is approved for the non-surgical treatment of gallstones and is strongly inhibitory in our hands. However, the clinical data from three cases we report here shows no effect of short-term administration of chenodeoxycholic acid on HDV viral load in individuals chronically infected with HBV/HDV. Clearly, our small case series falls short of a formal clinical trial. A major effect of chenodeoxycholic acid on HDV viral load at the standard dose for the treatment of gall stones is somewhat unlikely given this observation. In the setting of established chronic infection the effect of a moderately potent entry inhibitor may be subtle or only become apparent after prolonged administration given that there may be a large pool of stably infected hepatocytes. Along these lines, somewhat discouraging results have recently been reported from a phase I monotherapy trial of the HCV entry inhibitor ITX-5061 given for up to 28 days in the setting of established chronic infection with the hepatitis C virus [29]. However, we have not determined serum levels of chenodeoxycholic acid and hence cannot rule out that a higher dose might have an effect. However, there is extensive pharmacokinetic data suggesting that administration of a specific biliary acid at the currently used doses does likely not change the total serum level of BA, but rather shifts the composition of the BA pool [30–32]. Thus, we may not have reached an inhibitory serum level of CDCA. However, a formal clinical trial with comprehensive pharmacodynamics would be needed to fully clarify this point.

In this paper we demonstrate that BA that are substrates of NTCP inhibit HDV cell entry. This occurs most likely through competition for binding to NTCP between BA and the large HBV surface protein that has been shown to mediate binding of HBV and HDV to NTCP. Supraphysiological concentrations of BA were required for potent inhibition of viral cell entry and primary BA were somewhat more potent inhibitors than secondary, tertiary and synthetic BA. In addition we show data suggesting that the cotransporting function of NTCP is not required for its abilty to serve as a virus receptor. Finally, we report three clinical cases of chronically HBV/HDV-coinfected individuals where the primary BA chenodeoxycholic acid that is inhibitory *in vitro* and approved as a drug for the treatment of cholesterol-rich gallstones failed to produce any discernable alteration in HDV viral load when administered orally for a limited duration and at concentrations currently used for the treatment of gall stone disease. Whether an antiviral effect can be achieved with higher doses is unclear.

## Supporting Information

S1 Fig Lentiviral gene transfer of human NTCP into HuH-7 cells. **(A)** Parental HuH-7 cells and HuH-7 cells transduced with pWPI/Bsd-hNTCP and kept under blasticidin selection were fixed and stained for NTCP expression (green). Nuclei were counterstained with DAPI (blue). **(B)** FACS staining of HuH-7/NTCP cells for NTCP (solid line). Controls: no primary antibody (dashed line) and unstained (shaded area).
(TIF)

S2 Fig Inhibition of HDV infection by BA quantified by qPCR. On day 5 after infection in the presence or absence of 200 µM of the indicated BA total cellular RNA was prepared and HDV and GAPDH RNA were quantified. The results represent the HDV/GAPDH ratio for each sample with the value determined in cells infected in the absence of BA or other inhibitors set to 100%. The mean +/- SD from an experiment performed in duplicate is shown.
(TIF)

S3 Fig Effect of BA on HDV infection of primary human hepatocytes. Primary human hepatocytes (PHH) were exposed to HDV containing supernatant in the presence or absence of 200 µM of different BA. preS1 peptide (368 nM) served as positive control. After 6 h cells were washed and repleted with regular media without virus, BA or drugs. After another 5 d cells were fixed and stained with a polyclonal antibody against HDV antigen (right hand images in each panel). Nuclei were counterstained with DAPI (left hand images). A representative of three independent experiments is shown.
(TIF)

S4 Fig Effect of BA on HDV replication using IF as a read-out. HuH-7 cells were co-transfected with plasmids pSVLD3 and pT7HB2.7. After the transfection procedure was completed media was exchanged and cells were maintained in media with our without 100 µM of the indicated BA for 7 days. Then supernatant was removed and cells were fixed and stained for HDV antigen (green). Nuclei were counterstained with DAPI (blue).
(TIF)

S5 Fig Effect of BA on HDV replication using qPCR as a read-out. HuH-7/hNTCP cells were infected with HDV. After 6 h media was exchanged and cells were maintained in media with our without 100 µM of the indicated BA for 7 days. Then total cellular RNA was prepared and HDV and GAPDH RNA were quantified by qPCR. The results represent the HDV/GAPDH ratio for each sample with the value determined in cells infected and maintained in the absence of BA set to 100%. The mean +/- SD from three independent experiment performed in duplicate is shown.
(TIF)

S6 Fig Effect of BA on HDV particle assembly and release. Supernatant from the cells shown in S4 Fig. was precipitated with PEG to separate HDV particles from BA in the supernatant. Precipitates were then resuspended in regular media and used to infect naïve HuH-7/hNTCP cells in the absence of BA. Cells were stained for HDV antigen (green) on day 5 post infection.
(TIF)
