# Supplementary figures and images for: Primary Biliary Acids Inhibit Hepatitis D Virus (HDV) Entry into Human Hepatoma Cells Expressing the Sodium-Taurocholate Cotransporting Polypeptide (NTCP)
Source: PLoS One. 2015 Feb 3;10(2):e0117152. doi: 10.1371/journal.pone.0117152 (PMC4315608; doi:10.1371/journal.pone.0117152)

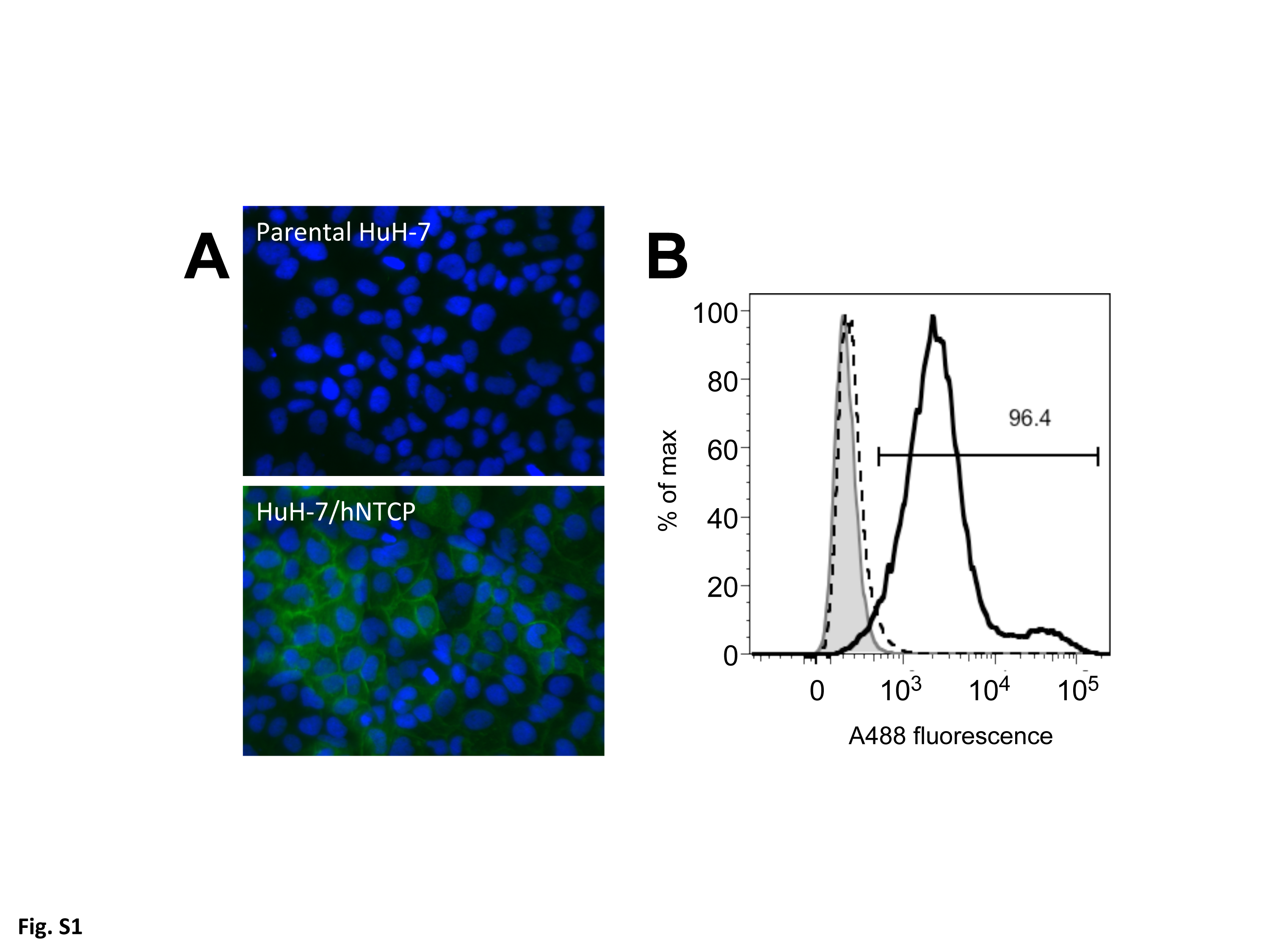

Supplement: S1 Fig — (A) Parental HuH-7 cells and HuH-7 cells transduced with pWPI/Bsd-hNTCP and kept under blasticidin selection were fixed and stained for NTCP expression (green). Nuclei were counterstained with DAPI (blue). (B) FACS staining of HuH-7/NTCP cells for NTCP (solid line). Controls: no primary antibody (dashed line) and unstained (shaded area). (TIF) [file pone.0117152.s001.tif]

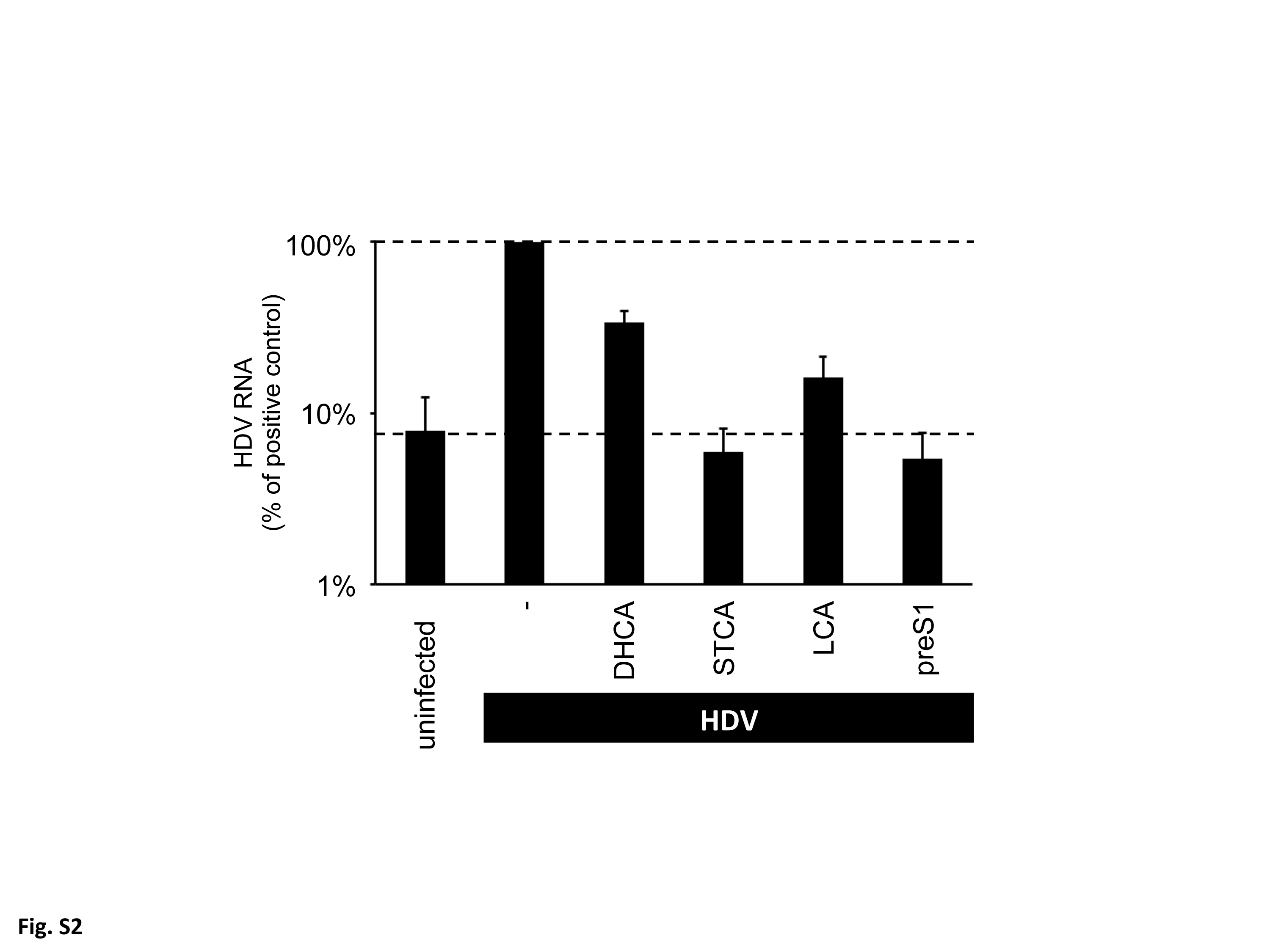

Supplement: S2 Fig — On day 5 after infection in the presence or absence of 200 µM of the indicated BA total cellular RNA was prepared and HDV and GAPDH RNA were quantified. The results represent the HDV/GAPDH ratio for each sample with the value determined in cells infected in the absence of BA or other inhibitors set to 100%. The mean +/- SD from an experiment performed in duplicate is shown. (TIF) [file pone.0117152.s002.tif]

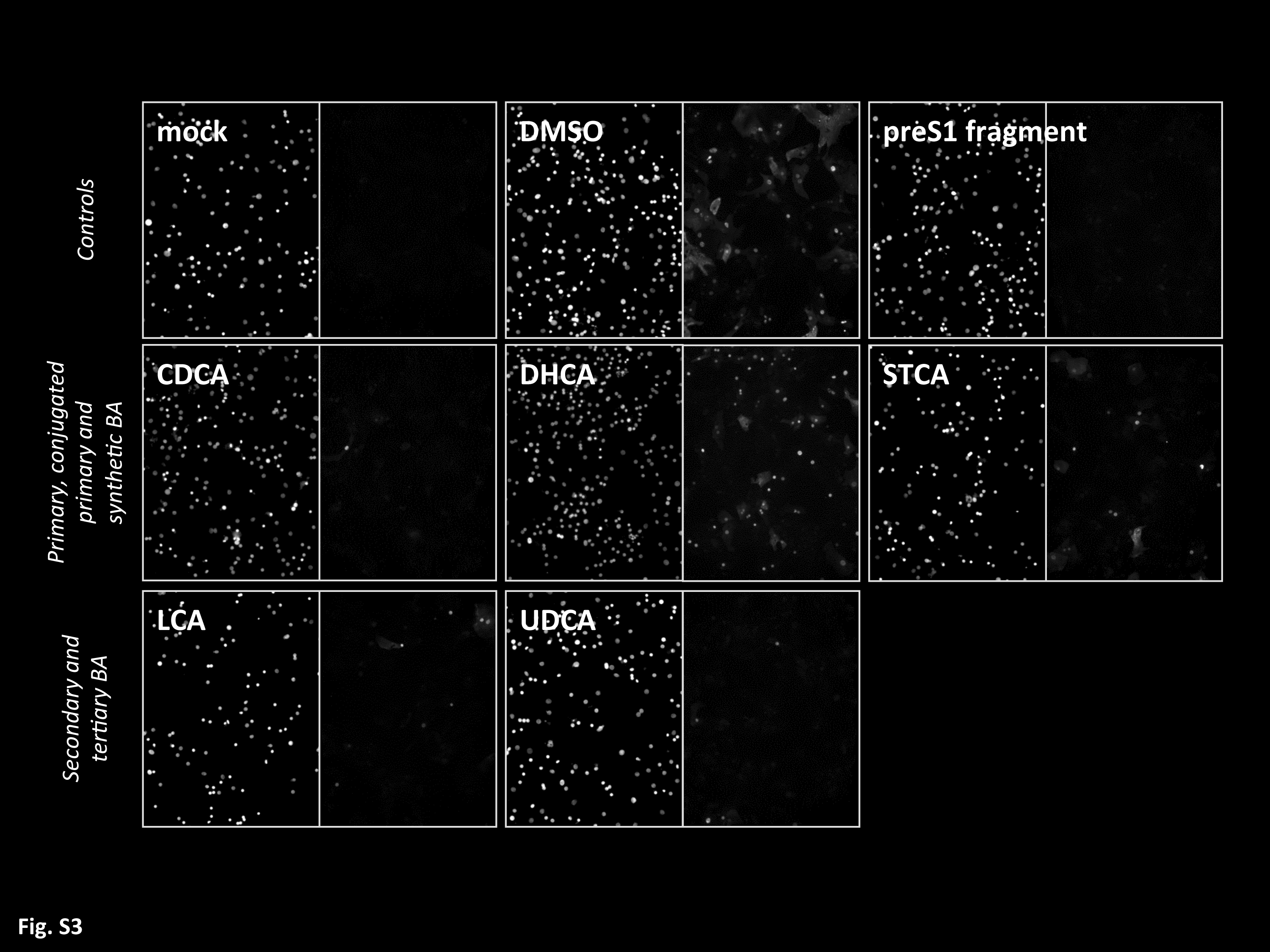

Supplement: S3 Fig — Primary human hepatocytes (PHH) were exposed to HDV containing supernatant in the presence or absence of 200 µM of different BA. preS1 peptide (368 nM) served as positive control. After 6 h cells were washed and repleted with regular media without virus, BA or drugs. After another 5 d cells were fixed and stained with a polyclonal antibody against HDV antigen (right hand images in each panel). Nuclei were counterstained with DAPI (left hand images). A representative of three independent experiments is shown. (TIF) [file pone.0117152.s003.tif]

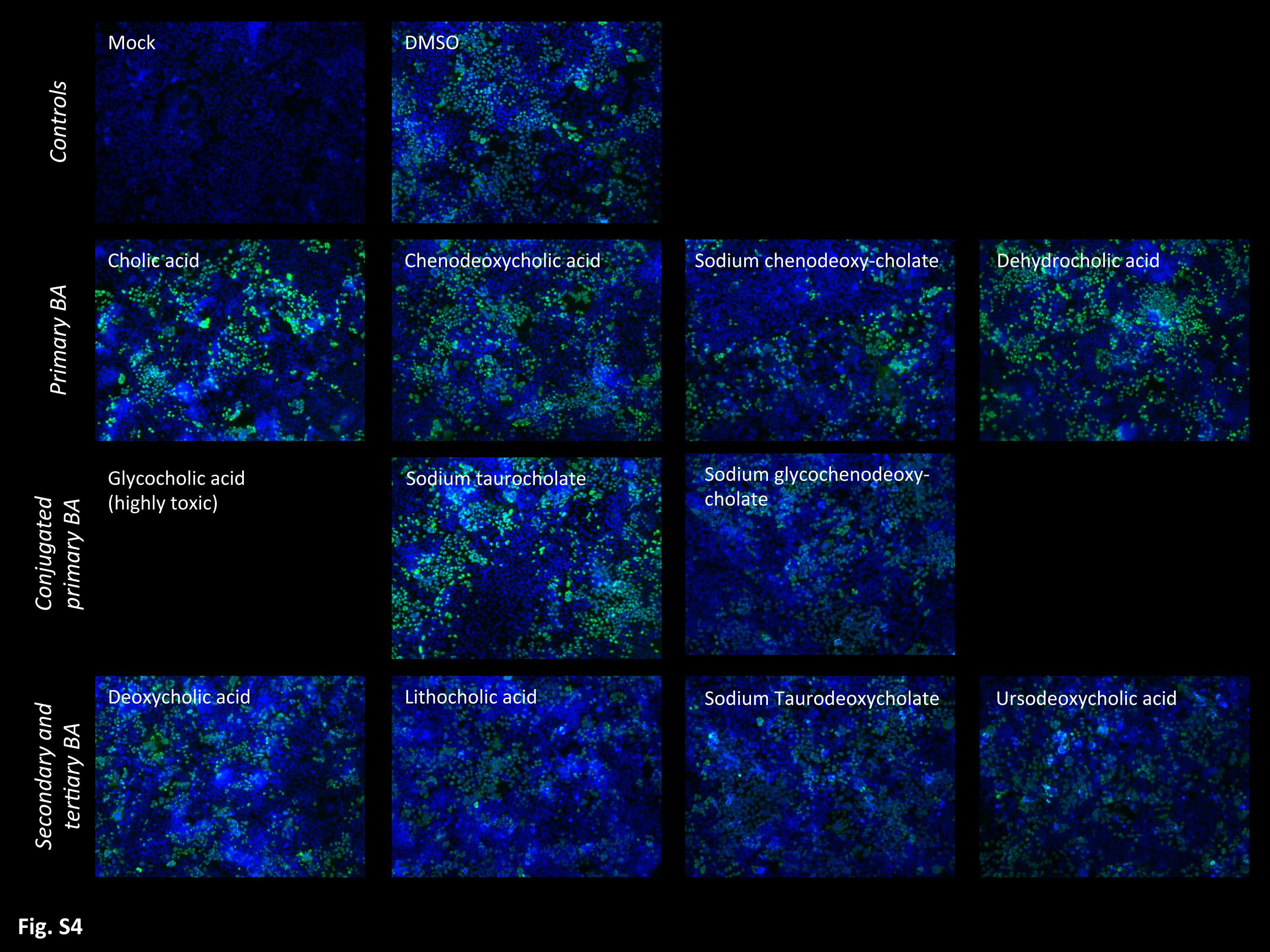

Supplement: S4 Fig — HuH-7 cells were co-transfected with plasmids pSVLD3 and pT7HB2.7. After the transfection procedure was completed media was exchanged and cells were maintained in media with our without 100 µM of the indicated BA for 7 days. Then supernatant was removed and cells were fixed and stained for HDV antigen (green). Nuclei were counterstained with DAPI (blue). (TIF) [file pone.0117152.s004.tif]

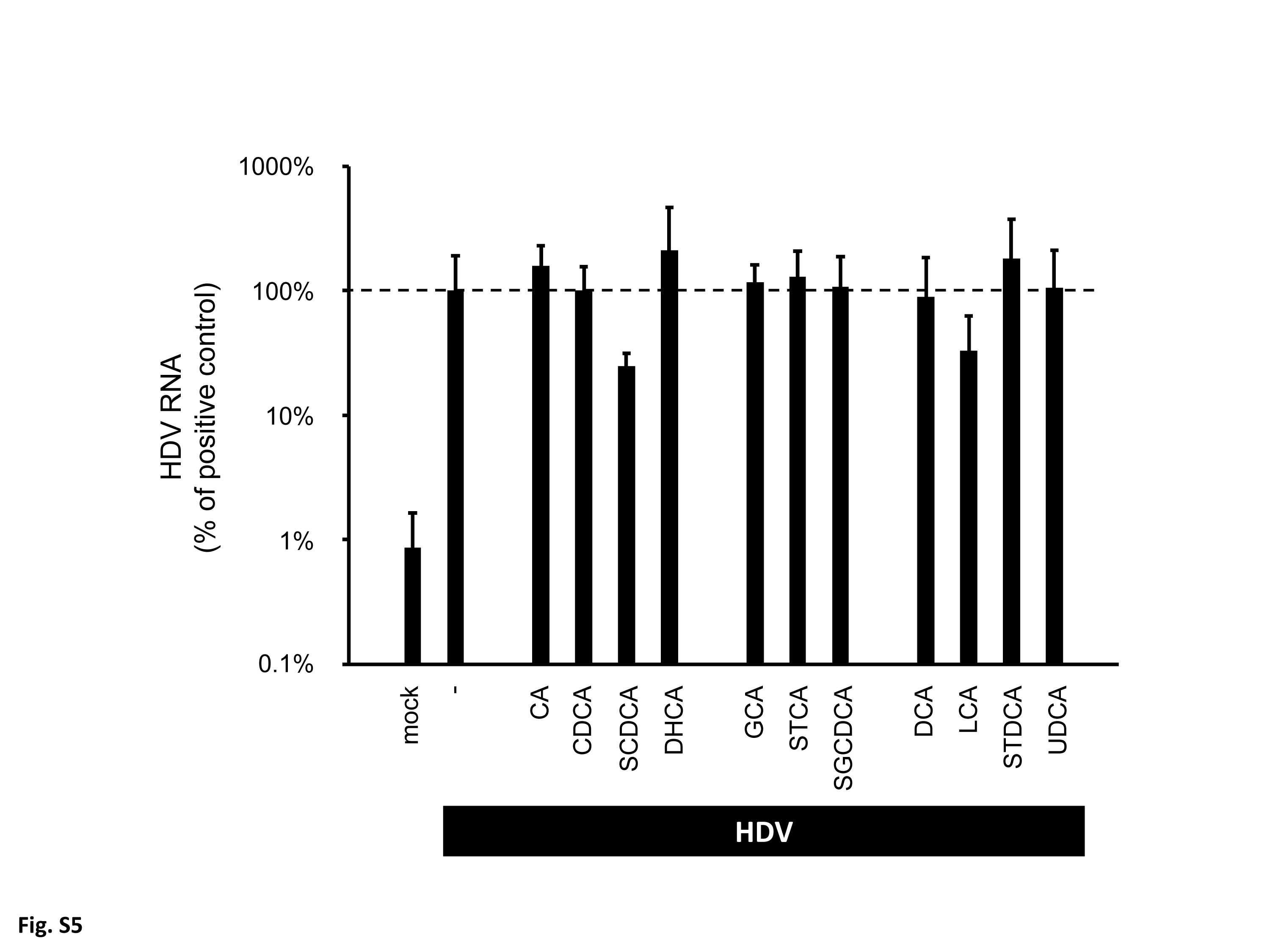

Supplement: S5 Fig — HuH-7/hNTCP cells were infected with HDV. After 6 h media was exchanged and cells were maintained in media with our without 100 µM of the indicated BA for 7 days. Then total cellular RNA was prepared and HDV and GAPDH RNA were quantified by qPCR. The results represent the HDV/GAPDH ratio for each sample with the value determined in cells infected and maintained in the absence of BA set to 100%. The mean +/- SD from three independent experiment performed in duplicate is shown. (TIF) [file pone.0117152.s005.tif]

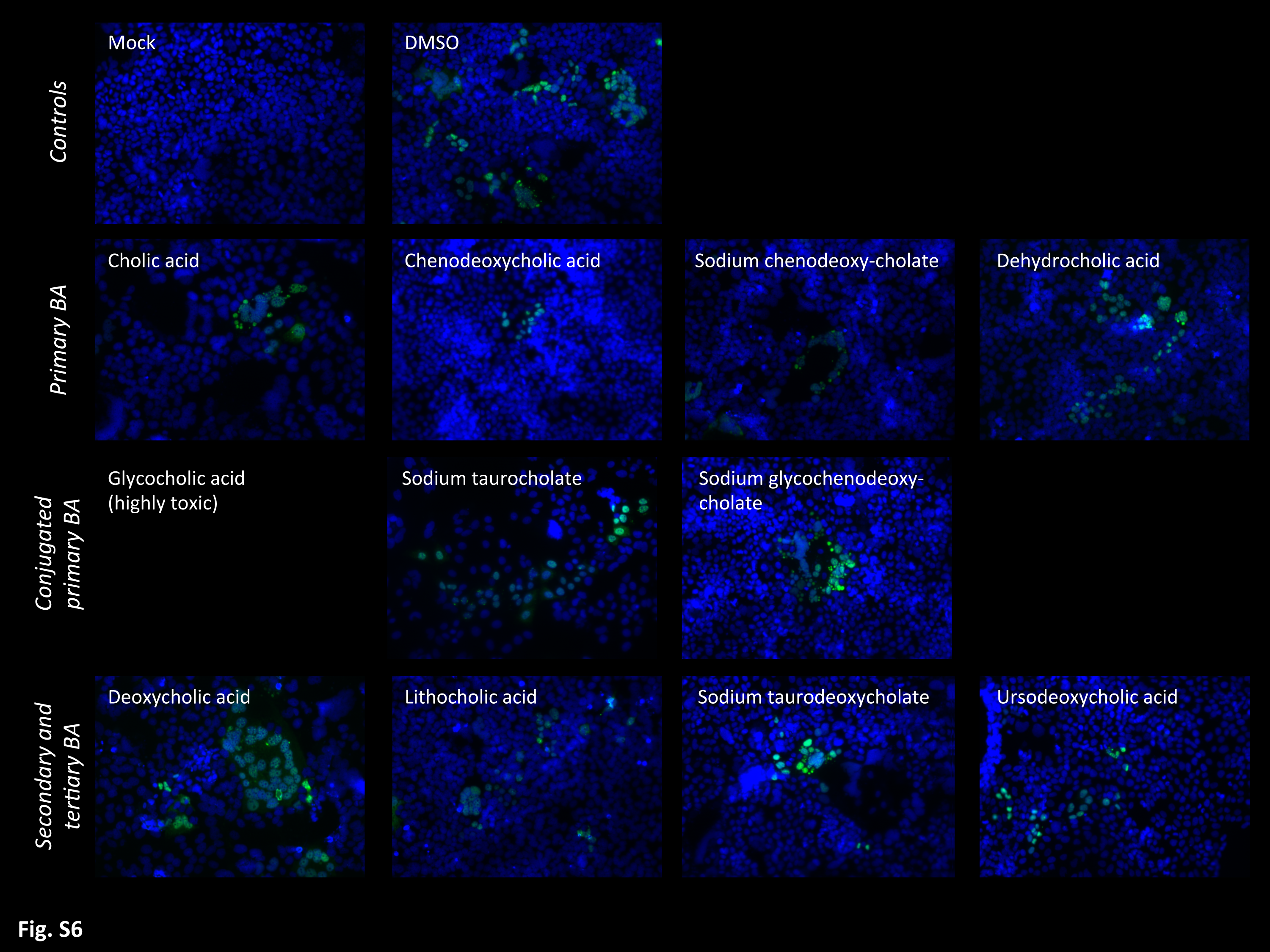

Supplement: S6 Fig — Supernatant from the cells shown in S4 Fig. was precipitated with PEG to separate HDV particles from BA in the supernatant. Precipitates were then resuspended in regular media and used to infect naïve HuH-7/hNTCP cells in the absence of BA. Cells were stained for HDV antigen (green) on day 5 post infection. (TIF) [file pone.0117152.s006.tif]
